# Typology of Dementia-Specific Care Units: A Nationwide Survey Study in Germany

**DOI:** 10.1093/geroni/igad062

**Published:** 2023-06-23

**Authors:** Johannes Michael Bergmann, Anna Louisa Hoffmann, René Müller-Widmer, Rebecca Palm

**Affiliations:** Deutsches Zentrum für Neurodegenerative Erkrankungen (DZNE) e.V., Witten, North Rhine-Westphalia, Germany; Witten/Herdecke University, Faculty of Health, School of Nursing Science, Witten, North Rhine-Westphalia, Germany; Deutsches Zentrum für Neurodegenerative Erkrankungen (DZNE) e.V., Witten, North Rhine-Westphalia, Germany; Witten/Herdecke University, Faculty of Health, School of Nursing Science, Witten, North Rhine-Westphalia, Germany; Deutsches Zentrum für Neurodegenerative Erkrankungen (DZNE) e.V., Witten, North Rhine-Westphalia, Germany; Witten/Herdecke University, Faculty of Health, School of Nursing Science, Witten, North Rhine-Westphalia, Germany; Witten/Herdecke University, Faculty of Health, School of Nursing Science, Witten, North Rhine-Westphalia, Germany

**Keywords:** Dementia, Long-term care, Multivariate analysis, Nursing, Residential facilities

## Abstract

**Background and Objectives:**

Dementia-specific care units vary in their organizational characteristics and are difficult to compare in empirical studies. Based on a representative sample of care units in German nursing homes, we present a typology of organizational characteristics focusing on dementia-specific care structures. We also examine the relationships between organizational types and the provision of nonpharmacological interventions for people with dementia.

**Research Design and Methods:**

Data were collected in a Germany-wide survey of a stratified randomized sample of 134 care units using a standardized questionnaire administered during telephone interviews with nursing home administrators or their representatives. The typology was developed based on a factor analysis of mixed data and a hierarchical cluster analysis.

**Results:**

We identified 4 types of care units: Dementia Care Units (DCUs; *n* = 40), Dementia Special Care Units (DSCUs; *n* = 17), Usual Separated Care Units (*n* = 58), and Usual Incorporated Care Units (*n* = 19). All care unit types clearly differed in their organizational characteristics. The specialization of DSCUs was agreed upon with cost bearers and included admission criteria, higher costs, and better staff conditions. Dementia Care Units without specialization did not have these characteristics. Three of seven nonpharmacological interventions were associated with the DSCUs and two with DCUs, but not with the other care unit types.

**Discussion and Implications:**

Researchers can use the typology to define and describe care units in empirical studies and improve the understanding and comparability of the context. A clear definition of care units also improves international comparisons.

## Graphical Abstract

**Graphical Abstract:**
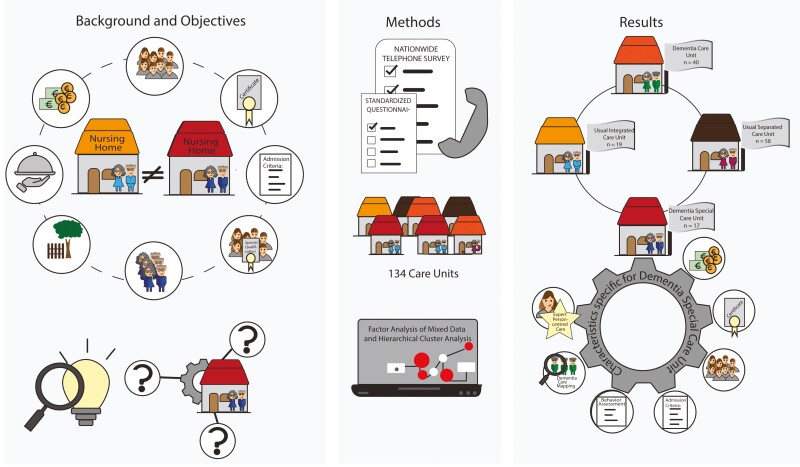

**Translational Significance:** The influence of the context of a care unit in health services research studies has rarely been explored. Therefore, it is valuable to develop a set of indicators that can be used to map differences between care units and define care unit types. Our developed typology provides an important baseline that enables researchers to describe the context of their intervention studies, and nursing home providers to implement innovative interventions in their context.

The definition of nursing homes and their organizational units—care units—is an important task for nursing home research. It is an international consensus that the definition of nursing homes and their care units is essential for high-quality evidence-based research, policy development, and quality improvement (Estabrooks et al., 2011; Sanford et al., 2015). Care units are considered “clinical microsystems” (Estabrooks et al., 2011) that frame the contextual conditions of care. Without respecting the context of nursing homes, research results are limited in their transferability, and the implementation of innovations may fail (Peryer et al., 2022).

In the 1990s, researchers in the United States attempted to define Dementia Special Care Units (DSCUs; Gold et al., 1991; Grant, 1998; Magaziner & Zimmerman, 1994). The researchers clarified that without a definition and clear description, it would not be possible to validly compare DSCUs with conventional care units. In particular, the dichotomy of DSCUs versus non-DSCUs must be overcome because this oversimplifies the diversity of their attributes. They recommended developing a typology of care units that acknowledges this diversity (Ohta & Ohta, 1988).

In 2014, we published a description of five types of care unit that was based on data from 103 care units in 51 nursing homes (Palm et al., 2014). We based the typology on three dichotomous a priori defined criteria (size, segregation of residents with dementia, and extra funding for additional staff resources) from the literature and known to be of relevance. From this, one could theoretically have formed eight types, of which only five could be realized in the sample with empirical cases. To identify further differences between these a priori types, additional tests were performed with external characteristics. In a subsequent analysis, we considered these previous results because they indicate that these types are associated with other structural characteristics; this implies that there are more complex relationships that can be considered for the development of care unit types (Bergmann et al., 2020). Using multiple correspondence analysis to develop an empirical typology on the same sample but including 19 variables related to staffing, work organization, building characteristics, etc., we described three complex care unit types. One care unit type was especially for people with dementia and corresponded to the international definitions of DSCUs.

Because the typologies published in 2014 and 2020 were based on data from a convenience sample that was not representative of the German population, we aimed to further develop our typology using current data from a randomized, stratified nationwide sample collected within a telephone-based survey. We were also interested in the association between different care unit types and the provision of nonpharmacological interventions provided in the care units. The typology development was guided by the following research questions according to our study protocol (Hoffmann et al., 2021):

1. Which contextual characteristics are associated with each other?
2. Which type of solution (formation) can be developed from the current study?
3. Which contextual characteristics are most significant for their respective types?
4. Are there differences between care unit types with respect to the implementation of nonpharmacological interventions to manage neuropsychiatric symptoms in dementia?

It was also our aim to compare our results with the previously developed typology and to confirm or reject our assumptions. In this paper, we report if we were able to validate our assumptions regarding dementia-specific care units and answer the research question:

5. To what extent are contextual characteristics of dementia-specific care units related to our a priori assumptions based on the previous empirical typology and literature?

In our study protocol, we defined for each variable and their categories the assumed type of care unit assignment. If empirical evidence was available, we based our assumption on this; if not, we based it on available theory (Hoffmann et al., 2021).

Regarding nonpharmacological interventions to manage neuropsychiatric symptom interventions, we expect that they are more often provided in dementia-specific care units.

## Research Design and Methods

### Design and Sample

We conceptualized the study as a nationwide survey study with a cross-sectional design and a stratified random sample of 160 nursing homes (Hoffmann et al., 2021). The study took place from February 2020 to May 2021 at the Deutsches Zentrum für Neurodegenerative Erkrankungen (DZNE) e.V., Site Witten.

The sampling frame was the total population of nursing homes in Germany. The random sample was drawn from a list of 11,658 German nursing homes that we purchased from a private data provider (Pflegemarkt.com, 2021).

### Stratified Randomization and Data Collection

The stratified random sampling is based on two variables from the purchased list: *federal state* and *availability of a DSCU.* To guarantee sample heterogeneity, we sampled the same number of nursing homes (*n* = 10) for each of the 16 German federal states. We chose the stratification variable *availability of a DSCU* to guarantee a distribution of nursing homes with and without a DSCU equal to the total population (20% with a DSCU, 80% without a DSCU). In case a nursing home supplied a DSCU, although they were sampled as a nursing home without a DSCU, they were nevertheless included in the DSCU group (or vice versa).

The list of nursing homes was randomly sorted, and nursing homes were contacted beginning at the top of the list. If the contacted nursing home neglected to participate, the next nursing home from the list was recruited until a total of 10 nursing homes per federal state agreed to participate. For each nursing home, we selected exactly one care unit, so that the data collection contains information at both the nursing home and care unit level. If a nursing home supplied a DSCU, data from that care unit were collected; otherwise, the care unit was randomly selected. Further details on the inclusion criteria and sampling procedure are reported elsewhere (Hoffmann et al., 2021). Stratified randomization was performed using the *sample()* function available in the base package of the statistical software R (R Core Team, 2022).

Data collection was conducted using computer-assisted telephone interviews and two standardized questionnaires. One researcher (A. L. Hoffmann) conducted all telephone interviews to ensure consistent interview quality. We developed a manual that specified each question to guarantee standardization. Parts of the questionnaire were newly designed and partly used in previous studies (Hoffmann et al., 2021). The questionnaire and data collection procedure were tested and adapted with four nursing home directors before data collection began. Recruitment and data collection took place between June and December 2020.

### Variables and Measurements

We assessed the data with two standardized questionnaires (nursing home and care unit). The nursing home questionnaire consisted of five items assessing organizational data. We additionally used data from the randomization list. This list contained the following information: urban location, federal state, and provider of the nursing home.

The care unit questionnaire assessed 34 items on the context: architecture (8 items), financing (5 items), professionals (6 items), residents (5 items), meals (3 items), and 7 items on dementia-specific interventions. An overview of the variables and their measurement is provided in Table 1. Detailed information on the questionnaire is reported in the study protocol (Hoffmann et al., 2021). Deviations between the study protocol and Table 1 can be explained as follows: from the nursing home questionnaire, we did not use the item “Existing full-time positions for nurses” because the item was highly associated with “Planned full-time positions for nurses.” We generated a staff ratio from the variables “Planned full-time positions for nurses” and the “Number of beds per nursing home” (RNRatio). From the care unit questionnaire, the variable “Numbers of residents in different care degrees” showed a high amount of missing data and was therefore excluded from the analysis; we combined the variables “Specialization of care unit,” “Type of specialization,” and “Inclusion criteria for admission to care unit” because the latter were subquestions (Special, Criteria). The variables “Number of rooms for residents” and “Availability of single rooms” were transformed to “percentage of single rooms.”

**Table 1.** Variables, Measurement, and Descriptive Results

Variable		Response	Short names	% (*N* = 134)/Mean (*SD*)
*Nursing home*				
Facility has a care unit specifically for people with dementia		No	DSCU 0	59% (79)
		Yes	DSCU 1	41% (55)
Number of care units per nursing home		Metric (total number)	Units	3.3 (1.6)
Urban location of the nursing home measured by the population		Less than 20,000 population	CityType 1	44% (59)
		20,000 to 100,000 population	CityType 2	23.9% (32)
		Over 100,000 population	CityType 3	20.9% (28)
		Over 1 million population	CityType 4	11.2% (15)
The 16 federal states of Germany		16 federal states	Baden-Württemberg, Bavaria, + 14 more federal states ( 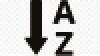 )	8.2% (11), 6.7% (9), 5.2% (7), 6.7% (9), 6% (8), 4.5% (6), 6.7% (9), 4.5% (6), 6% (8), 8.2% (11), 5.2% (7), 5.2% (7), 5.2% (7), 7.5% (10), 6.7% (9), 7.5% (10)
Provider of the nursing home		Nonprofit	Provider 0	56.7% (76)
		Profit	Provider 1	43.3% (58)
Number of beds per nursing home/planned full-time positions for nurses		Metric (staff ratio)	RNRatio	5.1 (1.4)
*Care unit*				
Architecture (organization and concept)				
Care unit is specially built for residents with dementia		No	Build 0	72.4% (97)
		Yes	Build 1	27.6% (37)
Care unit is architecturally separated from other units		No	Separate 0	12.7% (17)
		Yes	Separate 1	87.3% (117)
Care unit is protected by an exit control		No	Guarded 0	83.6% (112)
		Yes	Guarded 1	16.4% (22)
Care unit is on several floors		No	Floor 0	76.1% (102)
		Yes	Floor 1	23.9% (32)
Care unit has group living concept^a^		No	LivGroup 0	72.4% (97)
		Yes	LivGroup 1	27.6% (37)
Care unit has a directly accessible outdoor area		No	Outdoor 0	20.9% (28)
		Yes	Outdoor 1	79.1% (106)
Number of beds in care unit		Metric (total number)	Size	27.1 (11.8)
Percentage of single rooms		Metric (%)	SRoom	75.6% (27.6%)
Financing (regulation and allocation)				
Specialization of care unit agreed with cost bearers^b^^,^^c^		No	Special 0	84.3% (113)
		Gerontopsychiatric or closed care unit or DSCU/Other	Special 1	15.7% (21)
Subquestion on admission criteria for specialization	Admission criteria for residents agreed with cost bearers	No criteria for admission to care unit	Criteria 0	84.3% (113)
		Specialization of care unit agreed with cost bearers, but no extra admission criteria	Criteria 1	2.2% (3)
		Criteria for admission and specialization of the care unit	Criteria 2	13.4% (18)
Additional financing regulated by a special agreement		No	Finance 0	89.6% (120)
		Yes	Finance 1	10.4% (14)
Higher costs for residents compared to other care units		No	Costs 0	90.3% (121)
		Yes	Costs 1	9.7% (13)
Higher costs invested in additional staff (registered nurses)		No	StaffRN 0	89.6% (120)
		Yes	StaffRN 1	10.4% (14)
Professionals (structure and organization)				
Constant assignment of nurses (per care unit)		No	AssignRN 0	16.4% (22)
		Yes	AssignRN 1	83.6% (112)
Continuous presence of a registered nurse on night shift		No	NightPresRN 0	73.1% (98)
		Yes	NightPresRN 1	26.9% (36)
Continuous presence of a registered nurse on day shift		No	DayPresRN 0	9.7% (13)
		Yes	DayPresRN 1	90.3% (121)
Special qualification of head nurse in psychogeriatric care		No	JobQual 0	82.1% (110)
		Yes	JobQual 1	17.9% (24)
Full-time employment of the head nurse		No	FTHeadNurse 0	14.9% (20)
		Yes	FTHeadNurse 1	85.1% (114)
Head nurse is only responsible for one care unit		No	RespHN 0	75.4% (101)
		Yes	RespHN 1	24.6% (33)
Residents (care arrangements and diagnosis)				
Number of short-term care places in care unit		Short-term care places = 0	STC 0	71.6% (96)
		Short-term care places ≥1	STC 1	28.4% (38)
Number of residents with a medical diagnosis of dementia		Metric (%)	Dementia	69.6% (26.5%)
Number of residents who cannot be mobilized out of bed		Metric (%)	Mobile	3.5% (8%)
Number of residents with a court order for accommodation		Metric (%)	OrderA	10.3% (26.8%)
Number of residents with a court order for measures restricting their freedom—physical restraint		Metric (%)	OrderR	2.7% (5.7%)
Meals (preparation and service)				
Care unit offers opportunities to have dinner together		No	Dining 0	2.2% (3)
		Yes	Dining 1	97.8% (131)
Lunch is cooked in the kitchen of the care unit		No	Mealprep 0	88.1% (118)
		Yes	Mealprep 1	11.9% (16)
Meal service		Lunch on a tray	Mealserve 1	6% (8)
		Lunch is prepared on plates by staff	Mealserve 2	84.3% (113)
		All meals (breakfast, lunch, and dinner) are served homestyle on the table	Mealserve 3	9.7% (13)
Nonpharmacological dementia-specific interventions on the care unit				
Psychotropic drugs are regularly evaluated by internal quality management		No	Drugs 0	66.4% (89)
		Yes	Drugs 1	33.6% (45)
Dementia-specific instruments are used to assess pain		No	Pain 0	14.9% (20)
		Yes	Pain 1	85.1% (114)
Dementia-specific instruments to are used to assess behavior		No	Behavior 0	73.1% (98)
		Yes	Behavior 1	26.9% (36)
All staff members are obligated to be trained in person-centered care		No	Training 0	62.7% (84)
		Yes	Training 1	37.3% (50)
One staff member is an expert on person-centered care		No	Expert 0	80.6% (108)
		Yes	Expert 1	19.4% (26)
Dementia Care Mapping (DCM) is conducted once a year by a person not employed in the care unit		No	DCM 0	86.6% (116)
		Yes	DCM 1	13.4% (18)
Music therapy is offered once a week by a trained music therapist		No	Music 0	90.3% (121)
		Yes	Music 1	9.7% (13)

*Notes*:

^a^Group living concept means that the care unit is divided into small groups that share a living room and kitchen.

^b^Specialization means nursing home providers and the different cost bearers (statutory long-term care insurance and the municipalities) agreed that the care unit has a specialization (e.g., gerontopsychiatry, dementia).

^c^Cost bearers are the state social welfare agencies (municipalities) and the statutory long-term care insurance. Statutory long-term care insurance covers approximately one third of the whole costs. Either nursing home residents must pay for the missing part themselves or the state social welfare agency pays for them.

DCSU = Dementia Special Care Unit.

### Statistical Analysis

To investigate associations, we applied factor analysis of mixed data (FAMD) with hierarchical clustering (HC). FAMD calculates a set of eigenvalues and corresponding eigenvectors by analyzing categorical and metric variables in one single analysis. The first principal component (line of the best fit) represents the direction of maximum variance in the data set. The eigenvector is the direction of this line, whereas the eigenvalue informs about variance.

FAMD was used as a preprocessing technique to subsequently perform a clustering method on the principal components (Husson et al., 2017). This was done in a second step by applying the HC method to aggregate clusters (Blasius & Greenacre, 2014; Le Roux & Rouanet, 2010). Through this process, we identified the appropriate cluster structure of care units and defined the contextual characteristics that contribute to the cluster model. Subsequent to the HC, we calculated a test value (v test) to measure the extent to which the categorical and metric variables correspond with the identified clusters (Lebart et al., 1995). To validate the quality of the result in the last step, we used silhouette width as the internal cluster validation measure (Kassambara & Mundt, 2017).

Twenty-eight variables contributed to the definition of types (active variables), and nine variables (passive variables) were used to test differences between types. The active variables were used to calculate the eigenvalues and their corresponding eigenvectors, and the passive variables were used for the test of significance. As passive variables, we defined the seven nonpharmacological interventions, “Provider of the nursing home” (Provider) and the subquestion about “Admission criteria for residents contractually regulated with cost bearers” (Criteria). These variables were defined as passive because they are not constituent characteristics of the care unit types but are presumed to be related (subject to statistical testing) to their service structure. When clusters of care units stem from active variables, the v test can only be applied satisfactorily to passive variables (i.e., which were not used to determine the clusters), but they are also calculated for the active variables for information (Husson et al., 2017).

To impute missing values of a mixed data set with categorical and metric variables, the regularized iterative FAMD algorithm was used as a preliminary step before performing FAMD on an incomplete data set (Josse & Husson, 2016). For statistical analysis, we performed FAMD and HC using R statistical software (Lê et al., 2008; R Core Team, 2022). The data set and R code are available from the Zenodo repository (Bergmann et al., 2022). In October 2018, the ethical committee of the German Society of Nursing Science (Application number: 18-016) approved the research.

## Results

### Sample Description

In sum, we contacted 1,207 German nursing homes, representing 10.35% of the total population. Of these, 134 participated in the study, corresponding to a response rate of approximately 10%. Reasons for nonparticipation were stress due to the COVID-19 pandemic, staff shortages, and the resulting lack of time resources. We recruited nursing homes from all 16 German federal states. The median number of nursing homes surveyed per state was 8.5 (Min 6, Max 11). The median of care units per nursing home is 3 (Min 1, Max 8). For each of the 134 participating nursing homes, we collected data from exactly one care unit. In total, we included 55 care units (median per federal state 3, Min 1, Max 7) confirmed in the telephone interview as specifically for people with dementia (DSCU 1) for the DSCU stratum and 79 care units without specialization (median per federal state 4.5, Min 2, Max 9) confirmed in the telephone interview as not specifically for people with dementia (DSCU 0). We report the distributions for all variables described by the relative frequencies of the categorical variables and mean values of the quantitative variables in Table 1.

Out of the 4,958 statements that would have been necessary to complete the data set, 20 were missing. The missing data were confined to 15 care units. We replaced missing data with imputed data.

We excluded the variable “Care unit offers opportunities to have dinner together” from the cluster analysis because the category “Dining 0” has a very low frequency (less than 5%) and produces a lot of variance (disturbing influence) in the FAMD model. In this paper, we report the results of the HC only. Details of the results of the FAMD can be traced with the R code.

### Identified Care Unit Types: HC Results

Based on the results of the cluster analysis, we distinguish four care unit types:

- Type 1: Usual Incorporated Care Units (UICUs)
- Type 2: Usual Separated Care Units (USCUs)
- Type 3: Dementia Care Units (DCUs)
- Type 4: Dementia Special Care Units (DSCUs)

This cluster solution explains 31.3% of the total variance. The quality of this solution can additionally be determined by the average silhouette width of the clusters, which is reported in Supplementary Material.

Table 2 reports the results for the type description based on the categorical and metric variables. The categories and variables in Table 2 describing the four types are sorted in decreasing order according to their level of significance. The categories that are most significant for their respective types are therefore at the head of the table. The metric variables are equally ordered in descending order of significance but also differ by their positive or negative direction (test value). To present a summarized table as a result for the categories, we have decided to provide the percentages in the brackets only, as they are more descriptive for the distribution of a category. All listed categories and variables used to describe the four types satisfy the *p* < .05 requirement.

**Table 2.** Type Description

Usual incorporated care units (*N* = 19)	Usual separated care units (USCU) (*N* = 58)	Dementia care units (*N* = 40)	Dementia special care units (DSCUs) (*N* = 17)	Not significant
*Categorical variables* ^a^				
Separate 0 (82.4%, 73.7%, 12.7%)	DSCU 0 (70.9%, 96.6%, 59%)	DSCU 1 (70.9%, 97.5%, 41%)	Special 1 (81%, 100%, 15.7%)	LivGroup 0
NightPresRN 1 (50%, 94.7%, 26.9%)	Build 0 (59.8%, 100%, 72.4%)	Build 1 (70.3%, 65%, 27.6%)	StaffRN 1 (100%, 82.4%, 10.4%)	LivGroup 1
Floor 1 (50%, 84.2%, 23.9%)	NightPresRN 0 (57.1%, 96.6%, 73.1%)	Guarded 1 (59.1%, 32.5%, 16.4%)	Finance 1 (100%, 82.4%, 10.4%)	DayPresRN 1
DSCU 0 (24.1%, 100%, 59%)	Guarded 0 (51.8%, 100%, 83.6%)	Floor 0 (36.3%, 92.5%, 76.1%)	***Criteria 2*** *(83.3%, 88.2%, 13.4%)*	FTHeadNurse 0
***Training 0*** *(21.4%, 94.7%, 62.7%)*	***Criteria 0*** *(51.3%, 100%, 84.3%)*	StaffRN 0 (33.3%, 100%, 89.6%)	Costs 1 (92.3%, 70.6%, 9.7%)	FTHeadNurse 1
Build 0 (19.6%, 100%, 72.4%)	Special 0 (51.3%, 100%, 84.3%)	Finance 0 (33.3%, 100%, 89.6%)	Guarded 1 (40.9%, 52.9%, 16.4%)	STC 1
Mealprep 1 (43.8%, 36.8%, 11.9%)	StaffRN 0 (48.3%, 100%, 89.6%)	***Training 1*** *(44%, 55%, 37.3%)*	***DCM 1*** *(44.4%, 47.1%, 13.4%)*	Mealprep 0
***Expert 0*** *(17.6%, 100%, 80.6%)*	Finance 0 (48.3%, 100%, 89.6%)	Costs 0 (33.1%, 100%, 90.3%)	DSCU 1 (25.5%, 82.4%, 41%)	Mealserve 1
AssignRN 1 (17%, 100%, 83.6%)	Costs 0 (47.9%, 100%, 90.3%)	Separate 1 (33.3%, 97.5%, 87.3%)	***Behavior 1*** *(30.6%, 64.7%, 26.9%)*	Mealserve 2
Guarded 0 (17%, 100%, 83.6%)	Separate 1 (48.7%, 98.3%, 87.3%)	***Expert 1*** *(50%, 32.5%, 19.4%)*	Build 1 (29.7%, 64.7%, 27.6%)	Mealserve 3
***Criteria 0*** (16.8%, 100%, 84.3%)	***Expert 0*** *(50%, 93.1%, 80.6%)*		***Expert 1*** *(34.6%, 52.9%, 19.4%)*	***Provider 1***
Special 0 (16.8%, 100%, 84.3%)	DayPresRN 0 (84.6%, 19%, 9.7%)		Jobqual 1 (33.3%, 47.1%, 17.9%)	***Drugs 0***
***Pain 0*** *(30%, 31.6%, 14.9%)*	***Behavior 0*** *(51%, 86.2%, 73.1%)*		Outdoor 1 (16%, 100%, 79.1%)	***Drugs 1***
	AssignRN 0 (72.7%, 27.6%, 16.4%)		NightPresRN 1 (25%, 52.9%, 26.9%)	***Pain 1***
	Jobqual 0 (49.1%, 93.1%, 82.1%)		STC 0 (16.7%, 94.1%, 71.6%)	***DCM 0***
	Outdoor 0 (67.9%, 32.8%, 20.9%)		***Provider 0*** *(18.4%, 82.4%, 56.7%)*	***Music 1***
	RespHN 1 (60.6%, 34.5%, 24.6%)		AssignRN 1 (15.2%, 100%, 83.6%)	
	***Music 0*** *(46.3%, 96.6%, 90.3%)*		***Criteria 1*** *(66.7%, 11.8%, 2.2%)*	
			RespHN 0 (15.8%, 94.1%, 75.4%)	
*Metric variables* ^b^				
Size 39.2/27.1 (4.8)	OrderA 0.8%/10.3% (−3.6)	Dementia 92%/69.6% (6.4)	Dementia 92.5%/69.6% (3.8)	SRoom
Mobile 8.1%/3.5% (2.7)	Dementia 52.5%/69.9% (−6.5)	OrderA 19.7%/10.3% (2.7)	Units 4.6/3.3 (3.6)	RNRatio
Dementia 53.8%/69.6% (−2.8)		Units 3.8/3.3 (2.6)	OrderA 30.9%/10.3% (3.4)	OrderR
Units 1.2/3.3 (−6.2)		Size 22.6/27.1 (−2.8)		

*Notes*: Passive variables are shown in bold italics in the table.

^a^Categorical variables: the percentages in parentheses $\left(p_{1},\;p_{2},\;p_{3}\right)$ determine the statistical significance (*p* value). *p*_1_ specifies the percentage of care units possessing the corresponding category in the respective cluster. For example, 14 out of 17 care units with the category Separate 0 are included in the type “USCU,” i.e., $82.4\%\;\approx \;100*\frac{14}{17}.$*p*_2_ specifies the percentage of care units in the cluster possessing the corresponding category. For example, 14 out of 19 total care units in the type “USCU” have the category Separate 0, i.e., $73.7\%\;\approx \;100*\frac{14}{19}.\;$*p*_3_ specifies the percentage of care units possessing the corresponding category in the sample. For example, 17 out of 134 total care units have the category Separate 0, i.e., $12.7\%\;\approx \;100*\frac{17}{134}.$

^b^Metric variables: mean in type/overall mean (test value).

DCM = dementia care mapping.

We intentionally omitted from the type description the categories that negate the characteristics of care units (Category 0) for simplicity.

#### Usual Incorporated Care Units (*n* = 19, 14%)

Almost all care units (94.7%) in the UICU type continuously provide a registered nurse on the night shift (NightPresRN 1). In contrast, only 26.9% of care units in the total sample provide a registered nurse continuously on night shift. Thus, in this type alone, 50% of all care units (36) that continuously provide a registered nurse on night shift are represented. All care units of this type provide nursing staff permanently assigned to the unit (AssignRN 1). The care units of this type extend over several floors (Floor 1). Note that we found this type mainly in small nursing homes (<40 beds). We assume that small nursing homes are organizationally not divided into different care units, and therefore, the care unit in a small nursing home may be on more than one floor. Furthermore, it is evident that these care units more often prepare lunch in the kitchen of the care unit (Mealprep 1). More significant categories are displayed in Table 2. None of the passive variables indicated a characteristic of this care unit type.

Care units of this type provide more beds (Size) (39.2 in type vs 27.1 overall mean) and have a higher percentage of residents who are immobile (Mobile) (8.1% in type vs 3.5% in overall mean). The percentage of residents with dementia was lower (Dementia; 53.8% in type vs 69.6% overall mean). The care units of this type are located in nursing homes with fewer care units than in the whole sample (Units; 1.2 in type vs 3.3 overall mean).

#### Usual Separated Care Units (*n* = 58, 43%)

In this type, 98.3% of care units are architecturally separated from other units (Separate 1), whereas in the total sample, 87.3% (117) of care units are architecturally separated from other units. Thus, in this type alone, 48.7% of all care units that are architecturally separated from other units are represented. Another significant characteristic of the USCU type is that a head nurse is solely responsible for the care unit (RespHN 1). Other variables indicating the presence of a characteristic for this type were not significant. None of the passive variables indicated a characteristic of this care unit type.

The percentage of residents with dementia (Dementia; 52.5% in type vs 69.9% overall mean) and with an accommodation order (Order A; 0.8% in type vs 10.3% overall mean) is lower than in other care unit types.

#### Dementia Care Units (*n* = 40, 30%)

Thirty-nine of the care units in this type (97.5%) were designated by the facility managers as especially for people with dementia (DSCU 1). In contrast, in the total sample, 41% of care units were designated by facility managers as especially for people with dementia. Thus, in this type alone, 70.9% (40 of 55) of all care units that are especially for people with dementia are represented. Further significant characteristics of these care units are that they were built especially for residents with dementia (Build 1) and that they are protected by an exit control (Guarded 1). Accordingly, almost all care units are architecturally separated from other units (Separate 1).

Regarding the passive variables, two were significant: 55% (*p*_1_ = 44% category, *p*_3_ = 37.3% global) of the care units have a team that is trained in person-centered care (Training 1), and 32.5% (*p*_1_ = 50% category, *p*_3_ = 19.5% global) have one staff member that is an expert in person-centered care (Expert 1).

DCU characteristics also differentiate resident profiles. The percentage of residents with dementia was higher (Dementia) (92% in type vs 69.6% overall mean), and a higher percentage had an accommodation order (OrderA) (19.7% in type vs 10.3% overall mean) than those in other care unit types.

Most of the care units of this type have fewer beds than other care units (Size) (22.6 in type vs 27.1 overall mean). DCUs are often located in large nursing homes, which have more care units in total than other nursing homes (Units) (3.8 in type vs 3.3 overall mean).

#### Dementia Special Care Units (*n* = 17, 13%)

All care units of this type have a contracted specialization with cost bearers (Special 1). In contrast, only 15.7% (21) of care units in the total sample have a contracted specialization with cost bearers. Thus, in this type alone, 81% of all care units that have a contracted specialization with cost bearers are represented. The specialization is predominantly dementia-specific, which is why 82.4% of the care units were characterized by the facility managers as specifically for people with dementia (DSCU 1). The DSCU type covers all care units whose additional funding is regulated by a special agreement (Finance 1). The costs for the residents are higher compared to other care units (Cost 1); higher costs are invested in additional staff (registered nurses) (StaffRN 1). In addition, significant staff characteristics of the DSCU are that the head nurse has a special qualification in psychogeriatric care (Jobqual 1), and a registered nurse is always present during the night shift (NightPresRN 1). All nursing staff are permanently assigned to the care unit (StaffRN 1).

Further significant architectural characteristics of these care units are that they were built especially for residents with dementia (Build 1) and that they are protected by an exit control (Guarded 1). All care units of this type have a directly accessible outdoor area (Outdoor 1).

We note that 16 of 17 (94.12%) care units are architecturally separated from other units (Separate 1). This is not significant because the global value for the whole sample is 87.3% of care units that are architecturally separated from other care units.

Regarding the passive variables, five were significant:

(1) A total of 88.2% (*p*_1_ = 83.3% category, *p*_3_ = 13.4% global) of the care units in this type apply criteria for admission (Criteria 2), and 11.8% (*p*_1_ = 66.7% category, *p*_3_ = 2.2% global) have no admission criteria (Criteria 1).
(2) Most of the care units (82.4%; *p*_1_ = 18.4% category, *p*_3_ = 56.7% global) are provided by governmental, ecclesiastical, or nonprofit organizations (Provider 0).
(3) A total of 47.1% (*p*_1_ = 44.4% category, *p*_3_ = 13.4% global) of the care units conduct Dementia Care Mapping (DCM) once a year by a person who is not being employed in the care unit (DCM 1).
(4) A total of 64.7% (*p*_1_ = 30.6% category, *p*_3_ = 26.9% global) of the care units assess behavior regularly with dementia-specific instruments (Behavior 1).
(5) Slightly more than half of the care units (52.9%; *p*_1_ = 34.6% category, *p*_3_ = 19.4% global) have one staff member who is an expert in person-centered care (Expert 1).

The percentage of residents with dementia was higher (Dementia; 92.5% in type vs 69.6% overall mean) and with an accommodation order (Order A; 30.9% type vs 10.3% overall mean) than in other care unit types. DSCUs are often located in large nursing homes, which have more care units in total than care units in other nursing homes (Units; 4.6 in type vs 3.3 overall mean).

#### Comparison of the typology with a priori assumptions

In Table 3, we listed the characteristics that we assumed would apply to DCUs and if we could confirm this in our results. Of the 17 characteristics, we confirmed 14 for the DSCU type but only 5 for the DCU type. Four characteristics that we assumed to be characteristic of DSCUs could not be confirmed.

**Table 3.** Comparison of Assumed and Confirmed DSCU Characteristics

Assumed DSCU characteristics	DCU	DSCU
*DSCUs*		
Are built specifically for residents with dementia	✓	✓
Are separated from other care units	✓	
Are protected by an exit control	✓	✓
Have an accessible outdoor area	—	✓
Specialization is agreed with the cost bearer	—	✓
Apply admission criteria	—	✓
Are additionally financed, regulated by a special agreement/contract	—	✓
Have higher costs for residents with dementia	—	✓
Higher costs are invested in additional nursing staff	—	✓
Provide a nurse continuously on night shift	—	✓
Have a head nurse with special qualifications in psychogeriatric care	—	✓
Have a higher percentage of residents with dementia compared to other care units	✓	✓
Have a lower number of residents who cannot be mobilized out of bed compared to other care units	—	—
Have a higher number of residents with a court order for accommodation compared to other care units	✓	✓
Have a lower number of residents with a court order for measures restricting their freedom/physical restraints compared to other care units	—	—
Have a lower residents-per-registered nurse ratio^a^	—	—
Offer dining in the care unit	—	—

*Notes*: DCU = dementia care unit; DSCU = dementia special care unit.

^a^Measured at the nursing home level.

## Discussion

The aim of this study was to develop a representative dementia-specific typology of care units in German nursing homes and enhance our previously developed assumptions. Using cluster analysis techniques with subsequent validation of random data from 134 care units in 134 nursing homes, we identified four different types: two types of Dementia Care Units, Dementia-Specific Care Units and DCUs, and two different types of Usual Care Units, UICUs and USCUs. This is the first typology study in Germany that was developed from a representative sample of nursing homes and built on an existing typology.

**Figure 1.**
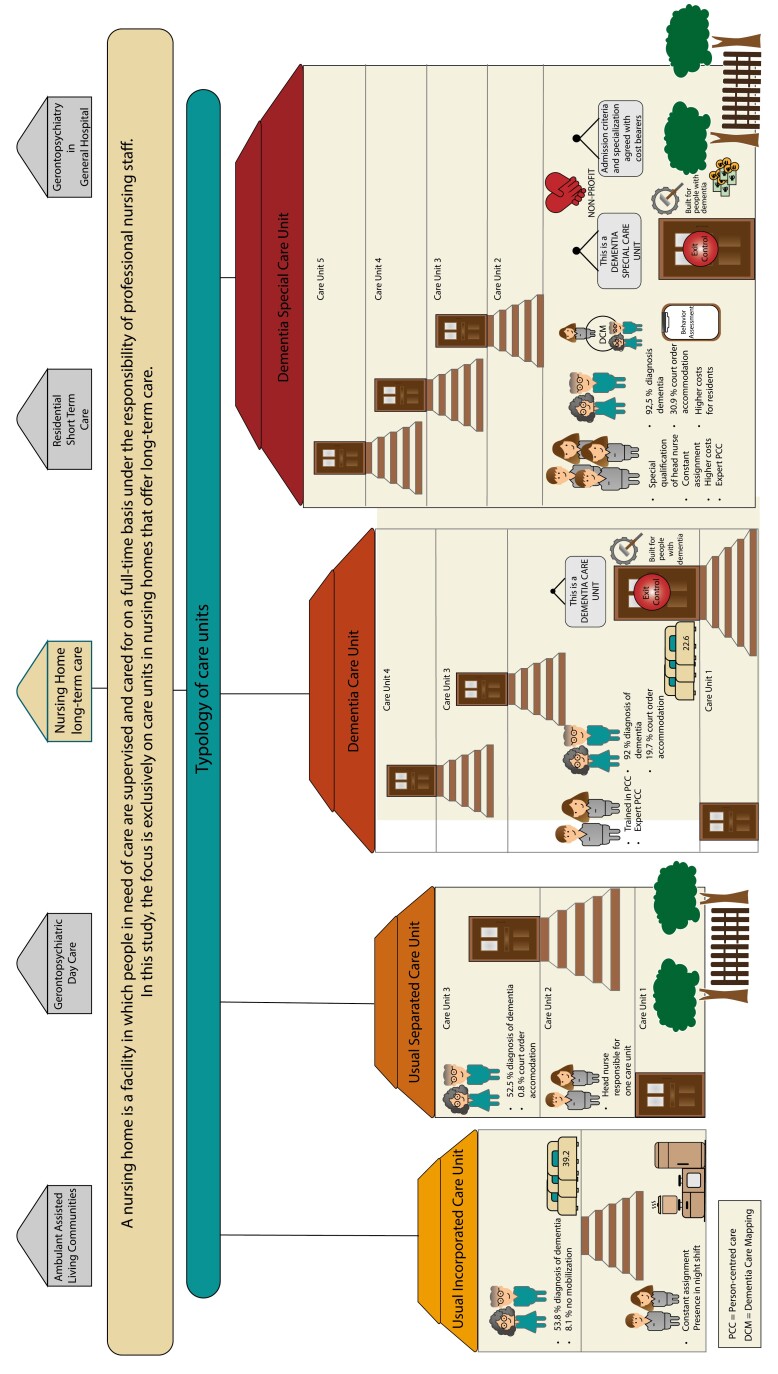
Illustration of care unit types in corresponding nursing homes for the German care system.

To give a visual impression of the results, we have graphically located our typology in the context of the German health care system in Figure 1. In contrast to services such as short-term care, gerontopsychiatric acute and day care in hospitals, and ambulant assisted living communities, which are based on different financing models, our typology refers exclusively to long-term care in nursing homes. We embedded the care units in the corresponding nursing homes, in order to reflect the actual size ratios of the facilities. The icons visualize the characteristics of the respective care unit types described in the results.

Our current analysis did not confirm the type of solution that we developed previously (Bergmann et al., 2020) but showed a more differentiated picture. Instead of one type of care unit for people with dementia, we distinguished two types (DSCU and DCU). We explain this as follows: types are defined by the combination of many characteristics, but some of these combinations can be traced to strong correlations to a few causal characteristics with great impact. This is evident in the distinction between the DSCU and DCU types by the systemic influence of having a specialization that is contractually agreed upon with the cost bearer. This contract with the cost bearer may also include other requirements of dementia-specific care unit that are evident in the strong associations (e.g., additional financing, costs, investment of costs in additional nursing staff, and admission criteria). Hence, this typology confirmed what we described in 2014 (Palm et al., 2014): that there is not one type of dementia-specific care unit in Germany and that not all care units that designate themselves as a DSCU have designated structural characteristics that usual care units do not have. The decisive factor here is that the specialization has to be contractually agreed upon with the cost bearers. Therefore, we could not confirm many of our assumptions for the DCU type but only for the DSCU type.

Regarding nonpharmacological interventions, we found that DCUs and DSCUs differ compared to both usual care unit types because we found only for the DCU/DSCU type associations. For the DCU type, both variables indicating the provision of person-centered care were characteristic: all staff members were trained in person-centered care, and one staff member was an expert. It should be noted, however, that although these variables are characteristic of this type, they are not found in all care units (55% train their staff in person-centered care, and 32.5% have an expert in person-centered care). Additionally, for the DSCUs, two nonpharmacological interventions were characteristic: the assessment of behavior with dementia-specific instruments (64.7%) and the conduction of dementia care mapping (47.1%). The fact that DSCUs are performing behavior assessments more often can be explained by the admission criteria of these units. What we see here is that although DSCUs have better structural conditions than DCUs, they do not seem to be superior to DCUs with regard to the use of nonpharmacological interventions.

Considering the two usual care unit types, we note that they differ significantly in the absence of many characteristics relevant to dementia-specific types, as indicated by the significance of the opposite category (e.g., Guarded 0, Build 0). We were also not able to reproduce the types we developed in the previous typologies (Bergmann et al., 2020; Palm et al., 2014), although some overlap was evident. We think that the reason for this is that our questionnaire did not describe the usual care units well because the focus of the study was the dementia-specific structures.

Several typology studies from the United States preceded this one. The first published was from Gold et al. (1991), followed by Grant (1998), Park et al. (2006), Degenholtz et al. (2006), and Corazzini et al. (2012). Although some of these studies have methodological limitations, such as single-state restrictions, no random sampling, or lack of primary data collection, and call for further research in this field, little research has emerged in the past two decades. Additionally, the typologies from the United States are hard to compare to the German typology because the contexts of nursing homes are very different. The most comparable study is from Grant (1998). Although more than 20 years old, the study still provides important findings. Grant identified six care unit clusters, of which one was the Dementia Care Unit type. This unit type had the most dementia care attributes and the highest proportion of residents with dementia. The Dementia Care Unit type had the following attributes that are comparable to our typology: locked doors, separation, staff training and constant nursing staff assignment, small unit size, provision of an outdoor area, and restrictive discharge policies. Similar to Grant (1998), we were able to describe a distinct type of care unit for people with dementia.

### Strength and Limitations

The strength of the study is random sampling, which ensured that any of the 11,658 nursing homes could have been selected. This is an important quality criterion to ensure the representativeness of a typology and is therefore also recommended for future studies (Gold et al., 1991; Park et al., 2006).

Some limitations of this study need to be considered. Due to stratification, we have a skew toward dementia-specific care units. Because we did not know about the reliability of our stratification criterion (“Nursing home provides a DSCU” vs “Nursing home does not provide a DSCU”) from the purchased list, we asked all contacted nursing homes initially about providing a dementia-specific care unit. If this was the case, then we prioritized collecting data from the dementia-specific care unit (DSCU 1). As a result, we oversampled nursing homes (41%) that offered a dementia-specific care unit. Because the primary purpose of this stratification procedure was to ensure that dementia-specific care units could be adequately accounted for in the typology, this is not problematic for the results. The list correction performed on the basis of the telephone survey (1,207 contacted nursing homes) made it possible to calculate an extrapolation for the proportion of dementia-specific care units in German nursing facilities of approximately 22.7%. Another limitation is the low response rate of selected nursing homes. Because data collection took place during the first wave of the COVID-19 pandemic in 2020 in Germany, nursing homes were burdened with restrictions, infection prevention, and high infection rates. This may explain why a greater number of nursing homes did not participate in our study. The high nonresponse rate may impede representativeness. Further limitations relate to the source of information and the variables collected. The data are self-reported by the respective facility managers and are therefore prone to be biased (e.g., social desirability). The variable RNRatio was calculated at the nursing home level and therefore only partially represents the differences between care unit types (nursing homes with and without dementia-specific care units).

## Implications

A significant implication grows out of methodological concerns in identifying appropriate experimental and control units when evaluating the effectiveness of DSCUs, respectively, interventions being implemented in these settings. In the future, researchers can use the typology to define the care units under study and to describe the context of an intervention. Hence, it may be possible to investigate whether an intervention shows better effects in one type than in others. In intervention research, an assignment to the typology helps researchers to control and evaluate the organizational context that influences implementation. This may also contribute to understanding the interplay between person- and task-focused factors and organizational and environmental factors. The culmination of these effects was identified to increase heterogeneity between nursing homes (Peryer et al., 2022).

Because we assume representativeness of the sample, a general assignment of care units is possible if the care unit approximates the described profile. We recommend that a care unit belongs to a type if it has at least three of the characteristics shown in Table 2. The more characteristics from the referring type a care unit has, the more reliable the assignment.

Furthermore, the typology can contribute to the international comparison and definition of care units. Future research and international exchange may lead to further development of sets of variables, their domains, and levels. The definitions of care unit types can help to monitor the care landscape of nursing homes and provide a clear view of changes over time. Following this, policy research can use the typology for extrapolations at the state and national levels.

## Supplementary Material

igad062_suppl_Supplementary_Materials

## Data Availability

The study is not preregistered as it is not a clinical intervention study. The study protocol has been published in advance. The data and the R code underlying this article are available in Zenodo repository, doi: 10.5281/zenodo.7215066
