# Supplementary material for: Typology of Dementia-Specific Care Units: A Nationwide Survey Study in Germany
Source: Innov Aging. 2023 Jun 23;7(6):igad062. doi: 10.1093/geroni/igad062 (PMC10433786; doi:10.1093/geroni/igad062)
Supplement: igad062_suppl_Supplementary_Materials [file igad062_suppl_supplementary_materials.docx]

**Online Supplementary Material**

***Quality of the Solution: Results of Silhouette Scores/Coefficients***


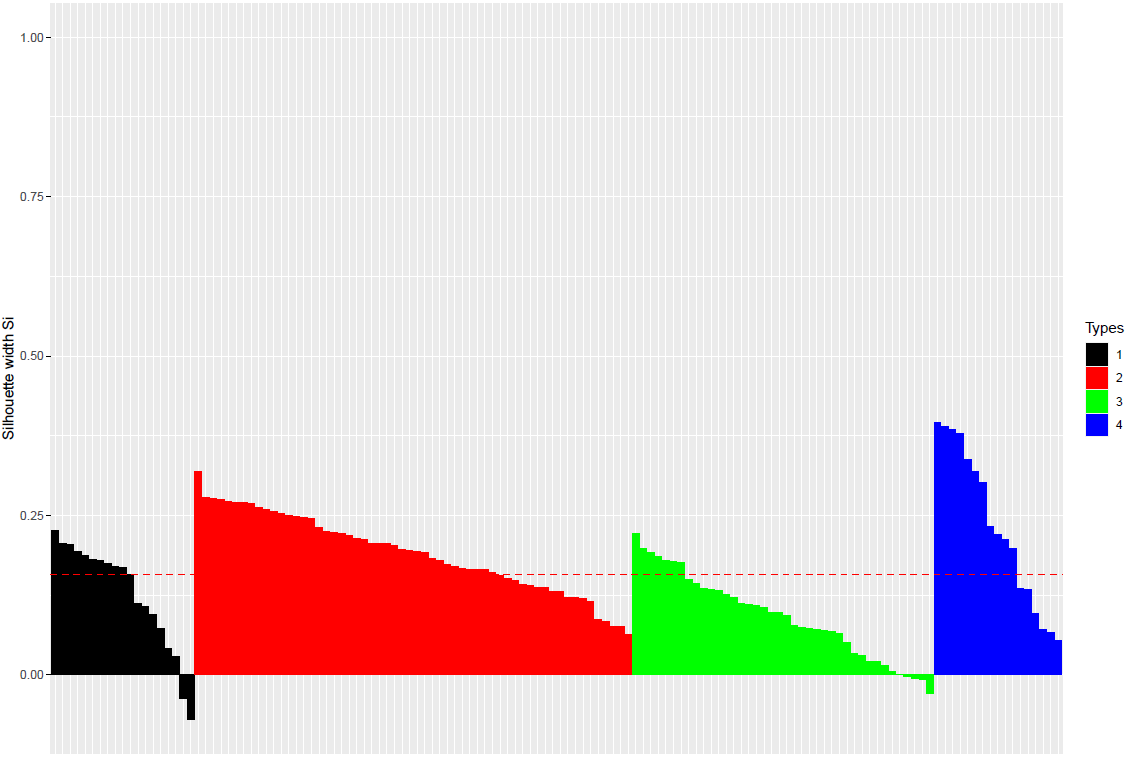


**Legend**

Types (f.l.t.r.):

1 Usual Incorporated Care Units average silhouette width 0.13 (black)

2 Usual Separated Care Units average silhouette width 0.19 (red)

3 Dementia Care Units average silhouette width 0.09 (green)

4 Dementia Special Care Units average silhouette width 0.23 (blue)

Overall average silhouette width: 0.16

**Description**

The silhouette plot in the Figure displays a measure of how close each care unit in one type is to care units in the neighboring types. Care units with a large positive silhouette width are very well clustered and are clearly separated from their nearest neighbor type. In contrast, care units with a negative silhouette width are probably placed in the wrong type. An analysis of the average silhouette width shows that the most discriminating type assignment is made for the DSCU type (blue = 0.23), followed by the USCU type (red = 0.19), the UICU type (black = 0.13) and the DCU type (green = 0.09). According to the interpretation of silhouette widths, the types DSCU and USCU do not contain wrongly assigned care units because they have all positive values.
